# Cooled Radiofrequency Ablation of the Lateral Femoral Cutaneous Nerve for Chronic Refractory Meralgia Paresthetica

**DOI:** 10.1007/s11916-025-01447-3

**Published:** 2025-12-29

**Authors:** Alaa Abd-Elsayed, Max Y. Jin, Austin P. Murphy, Lukas J. Henjum, Barnabas T. Shiferaw

**Affiliations:** 1 Department of Anesthesiology, University of Wisconsin School of Medicine and Public Health, Madison, WI USA; 2 University of Wisconsin School of Medicine and Public Health, 600 Highland Ave, Madison, WI 53792 USA

**Keywords:** Radiofrequency ablation, Chronic pain, Lateral femoral cutaneous nerve neuralgia, Meralgia paresthetica

## Abstract

**Purpose of Review:**

Chronic pain affects over 51 million people and is associated with a poor quality of life. Meralgia Paresthetica is a chronic pain condition resulting from injury to the lateral femoral cutaneous nerve. Radiofrequency ablation (RFA) is a minimally invasive treatment option that has demonstrated efficacy for several nerve targets. However, limited research has focused on RFA targeting the lateral femoral cutaneous nerve. This is the first study to date to investigate the outcomes of cooled RFA for the treating of refractory Meralgia Paresthetica.

**Recent Findings:**

Data were retrospectively collected from the electronic medical records of patients treated with cooled lateral femoral cutaneous nerve RFA for Meralgia Paresthetica between 2014 and 2025. Eleven cases involving seven patients were included in this study. VAS scores decreased from 5.91 ± 1.22 at baseline to 3.05 ± 2.64 at follow-up (*p* < 0.001). Patients in nine of the eleven cases reported improvement in pain, with an average reduction of 64.8%. Duration of pain relief after cooled RFA was 6.92 ± 3.23 months for six cases with this data available.

**Summary:**

Cooled lateral femoral cutaneous nerve RFA is effective for many patients with Meralgia Paresthetica and may be offered for refractory cases.

## Introduction

Chronic pain is a highly prevalent issue in the United States. About 20–21% of adults experience chronic pain, equating to over 51 million people [1]. With the recent COVID-19 pandemic, the number of people with chronic pain is only projected to rise due to the direct effects of the virus and the aftereffects of hospitalization [2]. Chronic pain has a severe, detrimental impact on a person’s quality of life. Specific aspects that are negatively affected include physical function, family and other relationships, work, sleep, and mood [3]. Furthermore, chronic pain is associated with increased risk for depression, anxiety, and substance abuse [4].

Meralgia Paresthetica is a specific type of peripheral neuralgia associated with the irritation, compression, or damage to the lateral femoral cutaneous nerve of the thigh [5]. This commonly results in sharp stabbing thigh pain and sensory abnormalities, including tingling and burning sensations or paresthesia. The lateral femoral cutaneous nerve is purely sensory and does not contain a motor component [6]. It provides sensation to the skin on the lateral aspect of the thigh from near the anterior superior iliac spine down toward the lateral knee. There are slight variations where sensory distribution can extend slightly to the anterior or posterior parts of the thigh. Pain can arise from various syndromes, trauma or injury, chronic irritation, or anatomic variations that leave this nerve vulnerable to compression [7–9].

There are numerous risk factors for developing Meralgia Paresthetica. Having hip or pelvic surgery can drastically increase the risk of developing lateral femoral cutaneous nerve complications that lead to numbness and tingling [10–13]. For total hip replacement surgeries, it has been estimated that the lateral femoral cutaneous nerve is injured in over 30% of cases [11]. Nonsurgical factors linked to increased risk of Meralgia Paresthetica include obesity or rapid weight change and diabetes mellitus [13]. Increased abdominal mass or changes in posture elevate tension across the inguinal ligament and increase extrinsic pressure on the lateral femoral cutaneous nerve. Meralgia Paresthetica is also frequently reported during late pregnancy or early postpartum. Diabetes is also a recognized risk factor via microvascular and connective tissue changes that heighten the nerve’s vulnerability to compression. Several clinical series and reviews note a higher prevalence of Meralgia Paresthetica among patients with diabetes and emphasize diabetes in the differential when sensory-only thigh dysesthesia is present [13, 14].

Radiofrequency ablation (RFA) is a minimally invasive procedure that uses heat generated by radiofrequency energy to target and disrupt nociceptive nerve fibers responsible for transmitting pain signals [15, 16]. Under ultrasound and/or fluoroscopic guidance, RFA denatures proteins in type C fibers responsible for transmitting burning pain [17]. Type Aα and Aβ fibers have higher survivability in high-temperature environments versus type C. This is beneficial because it can prevent numbness by keeping Aβ fibers intact while targeting type C nociceptive fibers, which are primarily associated with pain transmission [15]. Cooled RFA is an RFA technology that creates larger lesions without charring surrounding tissues [18]. Generally, RFA reduces reliance on pharmaceuticals, improving quality of life and reducing healthcare spending [19]. Due to the minimally invasive nature of this versatile procedure, which has demonstrated efficacy for many peripheral nerves throughout the body, it is crucial to investigate further the efficacy of utilizing RFA in treating lateral femoral cutaneous nerve pain [20–25].

Research focused on using RFA targeting the lateral femoral cutaneous nerve has been sparse to date [26, 27]. This retrospective analysis is the first study to investigate cooled RFA for treating Meralgia Paresthetica.

## Methods

This single-site, retrospective analysis received an IRB exemption from the University of Wisconsin IRB.

### Patient Selection and Diagnostic Blocks

Data were collected from electronic medical records of all patients treated with cooled lateral femoral cutaneous nerve RFA between 2014 and 2025. Patients in this study must have received cooled RFA targeting the lateral femoral cutaneous nerve as treatment for their chronic pain due to Meralgia Paresthetica. Before undergoing RFA, all patients needed to respond to two diagnostic nerve blocks (≥ 50% improvement). Diagnostic blocks were performed with 0.25% bupivacaine.

### Exclusion Criteria

Therapies distinct from cooled RFA, and thus excluded from this study, included chemical neurolysis, microwave ablation, cryoablation, and neurectomy. Patients were also excluded from the study if they did not have a baseline or follow-up pain score.

### Data Collection and Analysis

Pain was assessed with the 11-point validated Visual Analogue Scale (VAS), where higher scores signify greater pain. Specific data extracted included patient demographic information (age, sex, BMI, length of pain symptoms), RFA technique, pre- and post-operative VAS scores, duration of pain relief, and adverse events. Postoperative VAS score was obtained from the first follow-up available after the procedure, which was at least two weeks in duration. Each procedure was analyzed separately if patients underwent multiple cooled RFA procedures targeting the lateral femoral cutaneous nerve. Due to differences in BMI, age, and duration of pain symptoms during repeat procedures, demographic data were calculated for the cases (e.g., if there were “X” number of cases involving “Y” patients, the demographic data would be based on the “X” cases). Statistical analysis was completed using SPSS 30 software. A paired T-test was performed to assess the change in VAS scores from baseline to postoperative. A p-value less than or equal to 0.05 signified a statistically significant change.

### Procedure

Following informed consent, patients were sterilely prepped and draped in the supine position. Ultrasound was used to identify the lateral femoral cutaneous nerve at the lateral edge of the sartorius muscle, and 1% lidocaine was administered to anesthetize the skin. Under ultrasound guidance, a 17 g/50–100 mm/4–10 mm active tip needle was inserted. The exact dimensions of the needle varied based on provider preference. Upon needle insertion, testing was performed to ensure good sensory stimulation of the lateral thigh without motor stimulation of the lower extremity. Finally, 2mL of 2% lidocaine was injected through the needle, and cooled RFA was performed. The RFA settings were 80 °C tissue temperature for 150 to 165 s in the lesion mode. Following the procedure, a bandage was applied to the needle insertion site, and the patient was discharged home on the same day.

## Results

Eleven cases involving seven patients were included in this study (Table 1). One patient underwent the procedure three times, and two underwent it twice. Female and male patients were involved in six and five cases, respectively. Patients were a mean age of 52.55 ± 14.34 and a mean BMI of 34.60 kg/m^2^ ± 7.47. All patients were diagnosed with Meralgia Paresthetica, with six cases involving the right side and five cases involving the left. The mean duration of pain symptoms was 82.91 ± 82.67 months. All patients had previously failed conservative management strategies including physical therapy, chiropractics, psychotherapy, transcutaneous electrical nerve stimulation (TENS), oral analgesics, and massage therapy.

VAS pain scores decreased from 5.91 ± 1.22 at baseline to 3.05 ± 2.64 after the RFA procedure (*p* < 0.001). The average follow-up period for the postoperative score was 10.18 weeks (SD = 11.72). The mean percent improvement in pain was 53%, including two cases of patients who did not report any progress. For the nine instances where pain improvement was noted, there was a 64.8% reduction in pain. The mean duration of pain relief with cooled RFA was 6.92 ± 3.23 months for six patients, ranging from two weeks to nine months. Five of the six patients experienced relief for at least seven months. The three other patients who reported pain improvement did not have any available duration of relief (i.e., their final note stated the patient was still experiencing improvement). One adverse event of neuritis occurred and was resolved with a 5% lidocaine patch.

## Discussion

This is the first study investigating the use of cooled RFA of the lateral femoral cutaneous nerve for treating Meralgia Paresthetica. Our study found that most patients with chronic refractory Meralgia Paresthetica experience a significant reduction in pain when treated with cooled RFA targeting the lateral femoral cutaneous nerve. The average duration of pain relief was close to seven months. Of the two cases of patients who did not report an improvement in pain, one patient underwent the procedure bilaterally and only experienced relief on the left side.

### Comparison to Prior Literature

Previous literature, although sparse, has primarily focused on pulsed RFA. The largest study to date involved 11 patients with Meralgia Paresthetica treated with pulsed RFA [28]. 10 of the 11 patients experienced at least 50% relief by 12 months. Another study examined five patients who all reported significant pain relief, with at least an 87.5% improvement by their final follow-ups (ranging from 6 months to 2 years) [29]. Other case reports found similar results, with most reporting 100% relief [30–32], and one reporting 75% relief [33], at varying final follow-up periods up to two years.

One prior study has investigated continuous RFA for treating Meralgia Paresthetica in a case series involving six patients [34]. This study employed continuous thermal RFA, and patients reported a significant reduction in pain that started at 75.46% following the procedure, with sustained relief averaging nearly 40% at 6 months, with some patients reporting sustained relief after 12 months. All patients tolerated the procedure well in this study, and no adverse events were reported.

### Adverse Events

As with any interventional procedure, safety is always a concern. In our study, we observed one minor adverse event of neuritis, which was quickly resolved with a 5% lidocaine patch. In the prior studies that have utilized lateral femoral cutaneous nerve RFA, no complications were noted in most of them [28–30, 33, 34]. One case report reported an adverse event of numbness and paresthesia experienced by the patient [31]. The paresthesia resolved by the 1.5-month follow-up, while the numbness was still present at the three-month follow-up but did not interfere with function.

Overall, RFA has a generally favorable safety profile, with the most common adverse event being discomfort after the procedure, which usually resolves spontaneously [35]. In patients with pre-existing implanted devices, special considerations must be taken to mitigate the risks of electromagnetic interference by RFA, causing device complications [36]. Specific devices that may be affected by RFA include cardiac implantable electronic devices (e.g., pacemakers), spinal cord stimulators, intrathecal pumps, and deep brain stimulators.

### Review of Other Alternative Treatments for Meralgia Paresthetica

Alternative treatment options available for Meralgia Paresthetica are limited and lack thorough investigation [37]. Initial treatment involves conservative management with weight loss, loose clothing, physical therapy, topical agents, and oral analgesics [38, 39]. However, less than 70% of patients recover without further interventions [39]. Kinesio taping, topical capsaicin, and TENS are potential alternative non-invasive management strategies; however, the evidence is limited [40, 41]. Antidepressants are another pharmacologic treatment option that currently has limited evidence. Specifically, Duloxetine is a serotonin-norepinephrine reuptake inhibitor (SNRI) that has demonstrated reasonable benefit for the treatment of neuropathic pain [42], while other Selective Serotonin Reuptake Inhibitors (SSRIs) show weak effects overall comparatively [43].

Local injection therapies targeting the lateral femoral cutaneous nerve, typically involving a combination of local anesthetics and corticosteroids, represent a widely used treatment approach for Meralgia Paresthetica. These injections serve diagnostic and therapeutic purposes by temporarily disrupting pain transmission and reducing perineural inflammation [44]. However, evidence for injections as a primary treatment option has been mixed based on RCTs. Injections were found to offer similar benefits to TENS in one study [45], but were found to be no different than placebo in another [46]. Conversely, other non-RCTs have largely concluded injections to be effective in providing significant pain relief [41, 44, 47]. The drawbacks of local injection therapies are that the benefits are often short-lived, necessitating repeated injections which can be cumbersome and potentially increase risks for adverse events [48]. Therefore, while local injections remain valuable as a short-term minimally invasive option and diagnostic aid, they are generally insufficient as long-term solutions for chronic refractory cases.

Chemical neurolysis is another intervention similar to RFA that has been a potential treatment in several small studies. Ahmed et al. investigated a case series (*n* = 6) on the use of alcohol neurolysis for the treatment of intractable Meralgia Paresthetica. In this study, all patients demonstrated greater than 50% pain relief in all cases and decreased need for pharmacotherapy at the three-month follow-up [49]. An additional case study by Chen et al. (*n* = 1) showed similar findings with sustained relief six months following alcohol neurolysis of the lateral femoral cutaneous nerve [50]. However, given the increased serious risk profile of chemical neurolysis, this technique is not commonly employed. These severe risks include inadvertent spread to nearby tissues, skin necrosis, and motor paralysis. For this reason, chemical neurolysis is typically reserved for the treatment of terminal cancer patients with an expected life expectancy of less than one year [51].

In cases where conservative management and minimally invasive options fail, surgical decompression or neurectomy of the lateral femoral cutaneous nerve may be considered [47]. Surgery aims to relieve mechanical entrapment by releasing compressive structures such as the inguinal ligament or surrounding fascia [52]. Surgical intervention is effective for many patients, providing long-lasting results despite the initial pain [47, 52, 53]. However, surgery carries significant risk for complications and is accompanied by longer recovery periods. Furthermore, surgical options often come at higher costs for the patient [54]. For these reasons, surgery is typically reserved only for patients who have experienced no relief through repeated attempts with less invasive treatment options.

Given the limitations of alternative therapies and risks associated with surgical options, there remains a need for minimally invasive therapies that can provide long-lasting symptom control. Cooled radiofrequency ablation offers such potential by selectively targeting with controlled thermal lesions, combining efficacy with a favorable safety and recovery profile [16].

### Limitations

Limitations of this study include a small sample size and a retrospective design. Due to this study’s retrospective nature, we could not establish fixed follow-up periods for all cases.

### Future Directions

It is relevant to expand on patient risk factors associated with Meralgia Paresthetica and how they affect pain management outcomes with RFA treatment. This, in tandem with comparing the three RFA types with regard to Meralgia Paresthetica outcomes, would be beneficial. It is crucial to expand on which type of RFA treatment will be most effective for specific pathologies, especially regarding how effective they are at providing long-term or sustained relief from symptoms for over six months [55].

Another issue with RFA on nerves is regrowth of the nerve(s) [56], which can lead to repeat procedures. This presents an area of interest for research on developing novel RFA technologies because ideally, the nerve will not regenerate, and the treated nerve will no longer propagate pain signals. The development of such technology would reduce the need for repeat procedures, saving time and potentially reducing long-term patient costs.

Lastly, it is of interest to examine biomarkers for specific pain pathways regarding cooled versus pulsed and thermal RFA types. Biomarkers include various inflammatory cytokines, proteomics, and metabolites [57, 58]. Determining the mechanistic and physiological or anatomical properties of RFA modalities can lead to further treatment specification and awareness of which modality will produce the best durability of pain relief long-term.

## Conclusion

Cooled RFA can effectively manage Meralgia Paresthetica for many patients and may be offered when conservative treatments are unsuccessful. Additional research with larger samples and an RCT design remains critical to understand this intervention’s efficacy better. Furthermore, comparing outcomes for the three types of RFA technology used to treat Meralgia Paresthetica would be of great interest. Overall, RFA continues to be an essential topic in the treatment of chronic neuropathic pain due to its favorable safety profile and minimally invasive nature.

## Key References

- Lee, J.J.; Sohn, J.H.; Choi, H.J.; Yang, J.S.; Lee, K.H.; Do, H.J.; Lee, S.H.; Cho, Y.J. Clinical Efficacy of Pulsed Radiofrequency Neuromodulation for Intractable Meralgia Paresthetica. *Pain Physician* **2016**, *19*, 173–179.
  - ○This study has had the largest sample size (n=11) that examined pulsed RFA for treating Meralgia Paresthetica
- Abd-Elsayed, A.; Gyorfi, M.J.; Ha, S.P. Lateral Femoral Cutaneous Nerve Radiofrequency Ablation for Long-Term Control of Refractory Meralgia Paresthetica. *Pain Med*. **2020**, *21*, 1433–1436, doi:10.1093/pm/pnz372.
  - ○This is the only study that has examined continuous thermal RFA for treating Meralgia Paresthetica
- Abd-Elsayed, A.; Robinson, C.L.; Peters, T. Narrative Review of Radiofrequency Ablation Applications in Peripheral Nerves. *Ann. Palliat. Med*. **2024**, *13*, 89300–89900, doi:10.21037/apm-24-8.
  - ○This recent review examined what chronic pain conditions have been treated by RFA of various peripheral nerves

**Table 1:** Patient demographics

Sample size	11 cooled RFA procedures
Sex	Female − 6 Male − 5
Mean Age (SD)	52.55 years (14.34)
Mean BMI (SD)	34.60 kg/m^2^ (7.47)
Diagnosis	Right Meralgia paresthetica − 6 Left Meralgia paresthetica − 5
Mean duration of pain symptoms (SD) [range]	82.91 months (82.67) [12–240 months]

## Data Availability

Data is available upon request to the corresponding author.
